# Testing for sexually transmitted infections and blood borne viruses on admission to Western Australian prisons

**DOI:** 10.1186/1471-2458-9-385

**Published:** 2009-10-13

**Authors:** Rochelle E Watkins, Donna B Mak, Crystal Connelly

**Affiliations:** 1 Australian Biosecurity CRC, Curtin Health Innovation Research Institute, Curtin University of Technology, Perth, Australia; 2 Communicable Diseases Control Directorate, Western Australian Department of Health, Perth, Australia; 3 Health Services, Department of Corrective Services, Perth, Australia

## Abstract

**Background:**

Prison populations are known to be at high risk of sexually transmitted infections (STIs) and blood borne viruses (BBVs). In accordance with State health guidelines, the Western Australian Department of Correctional Services' policy is to offer testing for STIs and BBVs to all new prison entrants. This audit was undertaken to assess the completeness and timeliness of STI and BBV testing among recent prison entrants in Western Australia, and estimate the prevalence of STIs and BBVs on admission to prison.

**Methods:**

A retrospective audit of prison medical records was conducted among 946 individuals admitted to prison in Western Australia after the 1^st ^January 2005, and discharged between the 1^st ^January and 31^st ^December 2007 inclusive. Quota sampling was used to ensure adequate sampling of females, juveniles, and individuals from regional prisons. Main outcomes of interest were the proportion of prisoners undergoing STI and BBV testing, and the prevalence of STIs and BBVs.

**Results:**

Approximately half the sample underwent testing for the STIs chlamydia and gonorrhoea, and almost 40% underwent testing for at least one BBV. Completeness of chlamydia and gonorrhoea testing was significantly higher among juveniles (84.1%) compared with adults (39.8%; p < 0.001), and Aboriginal prisoners (58.3%) compared with non-Aboriginal prisoners (40.4%; p < 0.001). Completeness of BBV testing was significantly higher among adults (46.5%) compared with juveniles (15.8%; p < 0.001) and males (43.3%) compared with females (33.1%; p = 0.001). Among prisoners who underwent testing, 7.3% had a positive chlamydia test result and 24.8% had a positive hepatitis C test result.

**Conclusion:**

The documented coverage of STI and BBV testing among prisoners in Western Australia is not comprehensive, and varies significantly by age, gender and Aboriginality. Given the high prevalence of STIs and BBVs among prisoners, increased test coverage is required to ensure optimal use of the opportunity that prison admission presents for the treatment and control of STIs and BBVs among this high risk group.

## Background

Prison populations frequently engage in behaviours which put them at high risk for the transmission of sexually transmitted infections (STIs) and blood borne viruses (BBVs) [1,2]. The prevalence of STIs and BBVs is higher among prisoners than the general population, both in Australia [1,3] and elsewhere [4]. Control of STI and BBV transmission among high risk populations can contribute significantly to disease control in the wider population [5], particularly when disease transmission generally occurs in densely connected social networks [6].

Development of appropriate policy to address the public health needs of prisoners continues to present challenges in many countries [7-11]. Effective implementation of policies designed to improve prisoner health is also critical to the achievement of improved health outcomes among prisoners, and it is important that these policies and programs are adequately evaluated.

Department of Health guidelines in Western Australia [12] recommend opportunistic testing for STIs and BBVs among individuals who have a history of behavioural risk factors. In accordance with these guidelines, the Western Australian Department of Corrective Services' policy is to offer STI and BBV testing to all new prison entrants. To determine whether documented prisoner health assessment practices are consistent with STI and BBV testing policy, an audit was conducted to assess the completeness and timeliness of STI and BBV testing among prisoners in Western Australia, and estimate the prevalence of STIs and BBVs on admission to prison.

## Methods

### Setting

Western Australia has a total of 13 correctional facilities for adults, seven of which are within the greater metropolitan area of Perth. In the metropolitan area, prisoners on remand are managed at one of three prisons which provide separate services for adult males, adult females, and juveniles [13].

During the 2006-07 financial year, the daily average adult prisoner population in Western Australia was 3623 persons, with over 8180 adult prisoner admissions during this period. The daily average juvenile prisoner population in Western Australia was 139 persons, with a total of 1688 juvenile prisoner admissions during this period [14]. In 2007 approximately 8.5% of adult prisoners in Western Australia were female, and 42.9% were Aboriginal, which is defined here as inclusive of people of Aboriginal or Torres Strait Islander origin, who account for approximately 3.5% of the total Western Australian population [15].

### Sample

Adherence to STI and BBV testing policy was evaluated among a sample of prisoners admitted after 1st January 2005, and discharged between 1st January 2007 and 31st December 2007 inclusive. As all prison entrants are required to undergo an initial health assessment, no minimum duration of imprisonment was specified for inclusion in this audit. Prisoner records were sampled using data from the Department of Corrective Services' Total Offender Management System (TOMS) database, which maintains the records of all criminal offenders in Western Australia, and the Total Records and Information Management (TRIM) database, which contains scanned copies of discharged prisoners' paper medical records. Discharged prisoners were selected for inclusion in this audit because medical records are scanned into TRIM within four months of discharge, and therefore were available for examination at a central location.

Quota sampling was used to ensure that sufficient numbers of females, juveniles and prisoners from regional prisons were obtained to allow meaningful subgroup analysis by gender, age and Aboriginality. Among adult prisoners discharged from metropolitan correctional facilities, a target sample size of 200 male and 200 female prisoners was specified. Among juvenile prisoners discharged from metropolitan correctional facilities, a target sample size of 100 male and 100 female prisoners was specified. To ensure adequate representation of prisoners from regional prisons, a target sample size of 200 adult male prisoners discharged from the 6 regional correctional facilities for males was specified, and a target sample size of 100 adult female prisoners discharged from the 4 regional correctional facilities that accommodate female prisoners was specified. No regional correctional facilities exist for juvenile prisoners in Western Australia, and all juveniles audited were admitted to a single facility.

The study target sample size represents over 8% of adult prisoner admissions and over 11% of juvenile prisoner admissions in Western Australia per year. Eligible prisoners were sampled consecutively in reverse chronological order based on the date of discharge, commencing from the 31^st ^December 2007. The median sentence length among prisoners in Western Australia in 2007 was 2.7 years [15]. As such, sampling was performed in reverse chronological order from the last possible date of discharge to maximise the time between the eligible admission and discharge dates and decrease selection bias associated with the duration of imprisonment.

Juvenile females were the least commonly discharged sampling category, and due to the small available sample size, the sample of juvenile females was supplemented with eligible individuals whose records were not available on the TRIM database but were accessible for review at the juvenile remand centre on 9th June 2008.

### Data collection

Data were collected between the 28th April and the 9th June 2008. Examination of electronic medical records was restricted to documents relating to the initial health assessment and STI/BBV testing for the relevant prison admission. The TOMS database was used to retrieve eligible prisoners' identifiers (first and last initials and identification number) and demographic data (date of birth, sex, Aboriginality, date and location of admission and discharge). The TRIM database was used to identify the prison location where the health assessment was completed, documentation related to the uptake of STI and BBV testing, and results of testing based on the presence of an appropriate pathology report.

Medical and nursing staff employed by the Department of Corrective Services conducted the audit with the approval of the Department of Corrective Services' Director of Health Services. Data obtained were recorded on standardised data collection forms, and entered into a password protected Microsoft Access 2002 database. Processes used to ensure data quality during the audit process included the use of standardised forms for data extraction and entry, and verification of the accuracy of data entry prior to data analysis based on the comparison of original and extracted data, which was performed for over 10% of the audit sample.

As the audit's primary purpose was to evaluate and improve the quality of health care delivered to prisoners, it complied with requirements for a quality assurance study as defined by the National Health and Medical Research Council, and ethics approval was not required [16].

### Admission health assessment

Prison health assessment policies [17,18] require the assessment of a prisoner's health and wellbeing as soon as practicable following admission. All prisoners are required to be offered STI and BBV testing following admission to prison, and testing is to be completed within 28 days of admission. STI and BBV testing in Western Australian prisons is voluntary. Testing reviewed for this audit included nucleic acid tests for chlamydia and gonorrhoea, and serological tests for syphilis (i.e. STI tests), and serological tests for hepatitis B, C and HIV (i.e. BBV tests). A positive chlamydia or gonorrhoea test was one in which chlamydia or gonorrhoea nucleic acid was reported as having been detected. A positive syphilis, hepatitis C or HIV test was one in which antibodies to *Treponema pallidum*, hepatitis C or HIV were reported as having been detected. A positive hepatitis B test was one in which hepatitis B surface antigen was reported as having been detected.

### Data analysis

Data were analysed using SPSS (version 17.0, SPSS Inc.). Test coverage and disease prevalence results are reported by age group, sex and aboriginality due to the significant differences found between these subgroups. The Chi-square test of independence was used to test for associations between demographic characteristics and the completeness and results of STI and BBV testing. Pearson Chi-square statistics are reported using a two-sided significance level of 0.05. Odds ratios and their 95% confidence intervals (95%CI) are reported for significant associations.

## Results

### Sample

A total of 946 prisoners' records were audited (Table 1). Overall 57.5% of the sample (n = 544) were Aboriginal. Among adult prisoners, those sampled from regional prisons were more likely to be Aboriginal (76.8%) than those sampled from non-regional prisons (36.9%). Most (85.9%) prisoners audited were admitted to prison in 2007, with 11.2% admitted in 2006 and 2.9% admitted in 2005. Six adults and 31 juveniles were imprisoned for less than 24 hours, and 35 adults and 88 juveniles were imprisoned for less than 48 hours. Among adult prisoners who had a documented initial health assessment form on their electronic medical record (97.1%, 919/946), the health assessment was performed at the prison of admission for all prisoners apart from 3 prisoners, 2 of whom were admitted to regional prisons.

**Table 1:** Audit sample descriptive statistics by sex and age group.

**Subgroup**	**n**	**Mean age in years (range)**	**Mean number of days of imprisonment (range)**	**% Aboriginal**
Adult male	410	32.3 (18-66)	222.4 (0^†^-1019)	48.0
Adult female	302	32.7 (18-57)	107.1 (0^†^-912)	60.9
Juvenile male	122	15.6 (12-18)	7.8 (0^†^-176)	68.9
Juvenile female	112	15.7 (13-18)	22.1 (0^†^-334)	70.5

^†^duration of imprisonment of less than 24 hours

### Completeness of STI and BBV testing

**Figure 1:**
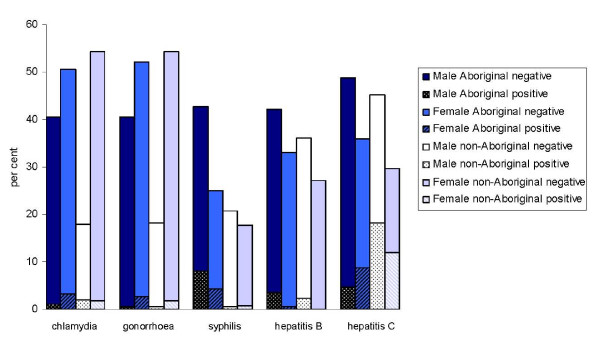
**Proportion of adults tested for STIs and BBVs (upper bar), and the proportion of the adults testing positive for STIs and BBVs (lower bar) by sex and Aboriginality**.

**Figure 2:**
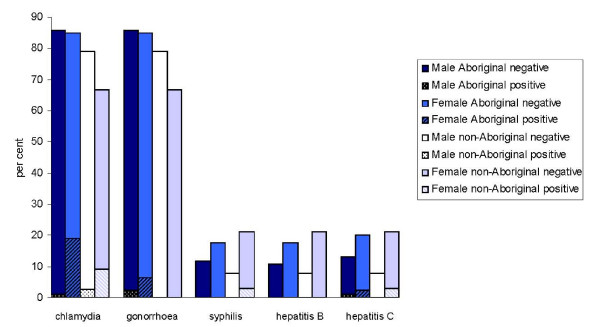
**Proportion of juveniles tested for STIs and BBVs (upper bar), and the proportion of juveniles testing positive for STIs and BBVs (lower bar) by sex and Aboriginality**.

STI testing for chlamydia and or gonorrhoea was documented for 50.5% of prisoners, and almost all of these (465/478, 97.3%) had undergone testing for both chlamydia and gonorrhoea. An additional 0.3% of prisoners refused testing, and 1.0% had STI tests ordered but no results were located. The completeness of STI testing for chlamydia and or gonorrhoea was significantly associated with age (p < 0.001, OR = 8.0 95%CI = 5.4-11.7) and Aboriginality (p < 0.001, OR = 2.1 95%CI = 1.6-2.7), with testing higher among juveniles (84.1%) compared with adults (39.8%), and Aboriginal (58.3%) compared with non-Aboriginal prisoners (40.4%). The completeness of STI and hepatitis B and C testing by sex and Aboriginality is summarised in Figures 1 and 2.

Syphilis test results were recorded for 24.2% of (229) prisoners audited. An additional 0.4% of prisoners refused testing, and 0.8% of prisoners had a syphilis test ordered but no results were located. Test coverage was significantly higher among Aboriginal (28.3%) compared with non-Aboriginal prisoners (18.7%) (p = 0.01, OR = 4.2 95%CI = 1.3-15.2). There was no significant association between syphilis test coverage and age (adults 27.4%, juveniles 14.5%; p = 0.08).

BBV testing was ordered or performed for either hepatitis B, hepatitis C, or HIV for 38.3% of (362) prisoners, and an additional 1.4% refused BBV testing. Among the prisoners who had BBV testing ordered or performed 79.0% had undergone testing for hepatitis B and 7.7% had a hepatitis B test ordered but no results were available; 91.2% had undergone testing for hepatitis C and 3.3% had a hepatitis C test ordered but no results were available; and 86.7% had undergone testing for HIV and 3.9% had a HIV test ordered but no results were available. Completeness of testing for BBVs was significantly associated with age (p < 0.001, OR = 4.6 95%CI = 3.2-6.8), and sex (p = 0.001, OR = 1.5 95%CI = 1.2-2.0), with testing higher among adults (46.5%) compared with juveniles (15.8%), and males (43.3%) compared with females (33.1%).

Test coverage and timeliness by age group and prison location is summarised in Table 2. Test coverage in the individual adult prisons studied ranged between 12.5% and 61.5% for STI testing, and 25.0% and 63.6% for BBV testing. Of the testing performed, the percentage of tests completed within 28 days of admission in the individual adult prisons studied ranged between 16.7% and 98.4% for STI tests and 29.0% and 85.7% for BBV tests.

**Table 2:** Test coverage and timeliness for sexually transmitted infections (STIs) and blood borne viruses (BBVs) by age group and location of prison of admission.

**Test**	**Metropolitan adults** **% (n)**	**Regional adults** **% (n)**	Juveniles* % (n)
STI coverage^†^	39.2 (163/416)	40.7 (120/295)	84.1 (195/232)
STI ≤ 7 days^††^	71.0 (110/155)	25.6 (30/117)	97.4 (185/190)
STI ≤ 28 days^††^	80.6 (125/155)	70.9 (83/117)	98.4 (187/190)
BBV coverage^‡^	47.2 (193/409)	45.5 (132/290)	15.8 (37/234)
BBV ≤ 7 days^‡‡^	9.1 (16/175)	15.6 (19/122)	69.7 (23/33)
BBV ≤ 28 days^‡‡^	43.4 (76/175)	63.9 (78/122)	97.0 (32/33)

*There are no juvenile correctional facilities in regional Western Australia

^†^excludes 3 refusals (1 metropolitan adult, 2 juveniles)

^††^proportion of those who had STI testing and information available on the time of testing

^‡^excludes 13 refusals (8 metropolitan adults, 5 regional adults)

^‡‡^proportion of those who had BBV testing and information available on the time of testing

### STI and BBV prevalence

Positive chlamydia test results were recorded for 7.3% (95%CI 4.9-9.7%) of the 466 prisoners tested. Among juveniles, females were significantly more likely to have positive chlamydia test results (18/89, 20.2%) than males (2/102, 2.0%; p < 0.001, OR = 12.7 95%CI = 2.8-55.6). Although positive chlamydia test results among Aboriginal females were approximately twice the rate of positive test results among non-Aboriginal females (Figures 1 and 2), there was no significant association between chlamydia test results and Aboriginality (p = 0.08) among females. Among males the trend was reversed, with non-Aboriginal males more likely to have positive chlamydia test results than Aboriginal males (p = 0.05).

Positive gonorrhoea test results were recorded for 3.4% (95%CI 1.8-5.0%) of the 470 prisoners tested. Although females had over twice the positivity rate of males (4.8% versus 1.8%), and Aboriginal prisoners had over twice the positivity rate of non-Aboriginal prisoners (4.1% versus 1.9%), gonorrhoea test results were not significantly associated with sex or Aboriginality (p = 0.07 and p = 0.2 respectively).

Positive treponemal test results indicating present or past syphilis infection were recorded for 11.8% (95%CI 7.6-16.0%) of the 229 prisoners tested. A non-Aboriginal female recorded the only positive syphilis result among the 33 juveniles tested. Among adults, Aboriginal prisoners were significantly more likely to have a positive syphilis test result than non-Aboriginal prisoners (18.5% versus 3.1%; p = 0.003, OR = 7.1 95%CI = 1.6-31.3).

Positive hepatitis B serology results were recorded for 4.5% (95%CI 1.2-2.1%) of the 286 prisoners tested. No positive findings occurred among juveniles. There was a significant association between sex and hepatitis B positivity among the 253 adults tested (p = 0.02, OR = 8.5 95%CI = 1.1-66.1), with 92.3% of the positive results occurring among males (Figure 1).

Positive hepatitis C serology or RNA test results were recorded for 24.8% (95%CI 20.2-29.5%) of the 330 prisoners tested. Among the 82 individuals with positive hepatitis C results, 53.7% had positive RNA test results, indicating active hepatitis C infection. Hepatitis C positivity was significantly associated with Aboriginality (p < 0.001, OR = 3.6 95%CI = 2.1-6.0), and age group (p = 0.04, OR = 3.0 95%CI = 1.0-8.7), with non-Aboriginal prisoners more likely to have positive hepatitis C test results (38.3%) than Aboriginal prisoners (14.8%), and adults more likely to have positive test results (26.6%) than juveniles (10.8%).

Among adults tested for hepatitis C, a positive test result was significantly associated with prison location among Aboriginal (p < 0.001, OR = 0.08 95%CI = 0.03-0.25), but not non-Aboriginal (p = 0.7) prisoners. Aboriginal prisoners discharged from regional prisons were less likely to be hepatitis C positive (4/100, 4.0%) than Aboriginal prisoners discharged from metropolitan prisons (21/62, 33.9%). Of the 314 prisoners tested for HIV infection, only 2 (0.6%, 95%CI 0.2-1.5%) had positive test results, and both were adults.

The joint evaluation of test coverage and the prevalence of STIs and BBVs is important for the estimation of the likely proportion of cases undetected, accounting for any targeting of testing, particularly if test coverage is not high. Figure 1 reveals comparatively lower rates of testing relative to apparent disease risk for hepatitis C among females and non-Aboriginal males, and syphilis among Aboriginal females. Comparatively lower coverage of chlamydia testing among non-Aboriginal males and juvenile non-Aboriginal females is also highlighted. Test refusal rates are not displayed in Figures 1 and 2 as they were low. Recorded rates of test refusal among the total sample were less than 0.5% for STI testing and less than 1.4% for BBV testing, with the greatest refusal rates on subgroup analysis among adult non-Aboriginal females for BBV testing (all ≤ 2.5%).

## Discussion

Prison health assessment processes provide an important opportunity to identify and treat STIs and BBVs among a high risk population. Our audit found that most tests for STIs and BBVs were performed for fewer than half of adult prisoners, and only a small proportion of prisoners who did not undergo testing had the refusal of testing documented on their electronic medical record. Few adult prisoners had very short durations of imprisonment that may have hindered the completion of STI and BBV testing, and compared with adults, STI test coverage was significantly higher among juvenile prisoners who often had a very short duration of imprisonment.

STI and BBV test coverage among adults audited was generally lower than that reported in a recent audit at a regional prison in Western Australia, where fewer than 45% of prisoners completed STI and BBV testing within 28 days of admission [3]. Differences in audit methods, including the audit of current practice, and the use of hard copy medical records which are not subject to a potential loss of information associated with the conversion to electronic format could in part explain the differences found in test coverage. However, together these studies indicate the need to improve the delivery of health services to prisoners on admission to prison.

Standardised assessment of prisoner health on admission is a widely endorsed strategy to support improved health care provision for prisoners [3,11,19,20]. Although documented rates of STI and BBV test refusal in our audit were low, the impact of undocumented refusals on test coverage was not able to be systematically assessed. Given that 55% of prisoners in Western Australia have had a prior adult imprisonment [15], undocumented test refusals associated with previous testing [21] may be an important unobserved influence on test coverage and disease prevalence estimates for chronic infections such as hepatitis B and C and HIV.

Additional guidelines may be needed to ensure that STI and BBV testing is offered to all prison entrants within 28 days of admission, that the requirements for the frequency of re-testing of prisoners on readmission are standardised, and that health assessment outcomes, including test refusal, are fully documented. Complete documentation of health assessment outcomes will enable more accurate monitoring of program effectiveness and the health needs of prisoners.

Our findings are consistent with international research which suggests that despite substantial evidence to support the effectiveness of prison health programs, considerable challenges confront the practical implementation of policies to improve prisoner health [22,23]. Successful implementation of recommended guidelines for improving prisoner health is critical to improved public health [7,23]. Further research is required to identify and address the barriers to more comprehensive health service delivery among prisoners, and enable the development of programs that promote the acceptance of testing, treatment, disease prevention and harm reduction strategies among prisoners and prison staff.

This study is unique compared with previous studies of prisoner health in Western Australia [1,3] in that we studied prisoners from both metropolitan and regional prisons, and sampled a large number of females, allowing comparisons of test coverage and disease prevalence by sex and Aboriginality. However, as sampling was stratified by age, sex and prison location to allow for meaningful subgroup analysis, comparisons of overall estimates of disease prevalence with the Western Australian prison population which is 91.5% male and 42.9% Aboriginal [15] may be misleading. The mean age of adult prisoners in this study was similar to the national prison population estimate of 35 years among males and 34 years among females [15], and 31 years among Western Australian prison entrants [1].

Our analysis found that prisoner sociodemographic subgroups with comparatively higher disease prevalence often had higher STI and BBV test coverage, although several areas of lower coverage relative to disease risk were highlighted; including hepatitis C among non-Aboriginal adult males and adult females, syphilis among Aboriginal adult females, and chlamydia among non-Aboriginal adult males and non-Aboriginal juvenile females. Lack of acknowledgement of the diversity among prisoner populations and their health needs has been reported elsewhere [7,24]. Our findings confirm differences in health needs by gender, ethnicity and age, and indicate a need for gender and culturally sensitive health services.

Disease prevalence estimates among prisoners in many cases reflect trends in disease notification rates among the general population, such as the high rates of chlamydia among juvenile females and female Aboriginal prisoners. However, the lower chlamydia testing rate among adult male prisoners is noteworthy given increased notification rates among males aged over 25 years [25]. As a result of this audit, given the observed high prevalence of chlamydia among juvenile females and the difficulties involved in providing follow-up clinical management, juvenile females are now offered empirical treatment for chlamydia at the time of testing [26]. The higher prevalence of gonorrhoea found among female prisoners compared with population disease notification data is consistent with the natural history of this infection, which tends to be asymptomatic and persistent in females versus symptomatic and self-limiting in males [12,25].

The prevalences of hepatitis B and C found in this audit were comparable with previous estimates among prisoners [1,3], and contrast with general population estimates which indicate that hepatitis C is more common among males and higher among Aboriginal people [25]. A high prevalence of hepatitis C among adult female prisoners is consistent with a high reported frequency of drug use among female prisoners [27]. We also found support for a geographical difference in the hepatitis C risk profile of Aboriginal prisoners, where Aboriginal prisoners in regional prisons had a significantly lower disease prevalence than both Aboriginal prisoners in metropolitan prisons and non-Aboriginal prisoners, as has been observed in South Australia [28].

Unfortunately RNA testing of prisoners who had hepatitis C antibodies was not performed routinely in Western Australia during the study period. As such, although test results enabled appropriate counselling regarding risk behaviours and the potential benefit of further testing or treatment, the proportion of prisoners who had active hepatitis C infection and would benefit from antiviral treatment was not known. Following this audit, routine collection of an additional blood sample for hepatitis C RNA testing was instituted, considering the high prevalence of hepatitis C antibody positivity in this population, and the benefits of antiviral treatment [29].

The HIV positivity rate found in this audit is equivalent to that found in the 2007 National Prison Entrants Survey [1], and considerably higher than the population-based age-standardised notification ratio for HIV of 3.6 per 100,000 population [25]. Higher rates of HIV in prisoner populations have been documented worldwide [5], and the current rate of HIV among Australian prisoners is likely to be higher than historical estimates [30].

To enable the local auditing of recent health assessment practices in metropolitan and regional prisons, we sampled recently discharged prisoners who had a relatively short duration of imprisonment. As such the ability to generalise our findings to the wider prisoner population in Western Australia, and in particular to prisoners admitted prior to 2006 and those with longer terms of imprisonment, is likely to be limited. Only a small proportion of prisoners were found to be missing an electronic record of their initial health assessment, and exclusion of these individuals from this audit is not likely to bias our findings, as test outcomes are unlikely to be related to the availability of a prisoner's electronic medical record.

Ongoing evaluation of prisoner health programs is needed to ensure effective policy implementation and improved prisoner health outcomes. The adoption of technologies such as electronic medical records [31] in prisons would enable more comprehensive centralised evaluation of program delivery and prisoner health outcomes based on representative samples of the prison population. Evaluation methods which improve the immediacy and completeness of feedback may also reinforce program effectiveness by increasing the perceived relevance and importance of program outcomes, and enhance the potential for audit findings to improve practice. Electronic medical record systems could also facilitate the identification of subpopulations that require targeted health interventions.

## Conclusion

Our findings identify limitations in the documented STI and BBV testing practices among prisoners in Western Australia, and confirm the importance of providing comprehensive assessment, treatment and counselling for this high risk population. Interventions are required to improve the coverage of STI and BBV testing among prisoners on admission to prison, and ongoing program evaluation is essential to ensure the effective implementation of policies designed to improve prisoner health. Deficiencies in prisoner health assessment practices represent missed opportunities to improve disease control in prisoners and in the wider population.

## Competing interests

The authors declare that they have no competing interests.

## Authors' contributions

DBM conceived and supervised the study. CC provided information on the Department Corrective Services' policies and practices, and organized access to the data. REW performed the data analysis and drafted the manuscript, and all authors read and approved the final manuscript.

## Pre-publication history

The pre-publication history for this paper can be accessed here:



## References

[B1] Butler T, Papanastasiou C (2008). National prison entrants' bloodborne virus and risk behaviour survey report 2004 & 2007.

[B2] Hellard ME, Hocking JS, Crofts N (2004). The prevalence and the risk behaviours associated with the transmission of hepatitis C virus in Australian correctional facilities. Epidemiol Infect.

[B3] Gilles M, Swingler E, Craven C, Larson A (2008). Prison health and public health responses at a regional prison in Western Australia. Aust N Z J Public Health.

[B4] Murray E, Jones D (2008). Audit into blood-borne virus services in Her Majesty's Prison Service. Int J STD AIDS.

[B5] Hellard ME, Aitken CK (2004). HIV in prison: what are the risks and what can be done?. Sex Health.

[B6] Ward H (2007). Prevention strategies for sexually transmitted infections: importance of sexual network structure and epidemic phase. Sex Transm Infect.

[B7] Fraser A, Gatherer A, Moller L (2009). Social justice, public health and the vulnerable: health in prisons raises key public health issues. Public Health.

[B8] Jurgens R, Ball A, Verster A (2009). Interventions to reduce HIV transmission related to injecting drug use in prison. Lancet Infect Dis.

[B9] Levy MH, Treloar C, McDonald RM, Booker N (2007). Prisons, hepatitis C and harm minimisation. Med J Aust.

[B10] Mackie P, Morling J (2009). Commissioning prison health: opportunities and challenges for creating a new prison public health system in Scotland. Public Health.

[B11] Spaulding AC, Weinbaum CM, Lau DT, Sterling R, Seeff LB, Margolis HS, Hoofnagle JH (2006). A framework for management of hepatitis C in prisons. Ann Intern Med.

[B12] Department of Health Western Australia (2006). Guidelines for managing sexually transmitted infections.

[B13] Department of Corrective Services (2008). Department of Corrective Services 2008 Handbook: A guide to services provided by the Department.

[B14] Department of Corrective Services (2007). Annual Report 2006-07.

[B15] Australian Bureau of Statistics (2007). Prisoners in Australia. ABS Cat No. 4517.0. Canberra.

[B16] National Health and Medical Research Council (2003). When does quality assurance in health care require independent ethical review? Advice to Institutions, Human Research Ethics Committees and Health Care Professionals.

[B17] Department of Corrective Services (2007). CC-02 Admission Assessment Process.

[B18] Department of Corrective Services (2007). CC-7 Blood Testing and Notification of Results.

[B19] Watson R, Stimpson A, Hostick T (2004). Prison health care: a review of the literature. Int J Nurs Stud.

[B20] Butler T, Spencer J, Cui J, Vickery K, Zou J, Kaldor J (1999). Seroprevalence of markers for hepatitis B, C and G in male and female prisoners--NSW, 1996. Aust N Z J Public Health.

[B21] Reniers G, Eaton J (2009). Refusal bias in HIV prevalence estimates from nationally representative seroprevalence surveys. AIDS.

[B22] Anonymous (2007). Putting policy into practice on prison health. Lancet Infect Dis.

[B23] Moller L, Gatherer A, Dara M (2009). Barriers to implementation of effective tuberculosis control in prisons. Public Health.

[B24] Rutherford M, Duggan S (2009). Meeting complex health needs in prisons. Public Health.

[B25] Department of Health Western Australia (2007). The Epidemiology of Notifiable Sexually Transmitted Infections and Blood-Borne Viruses in Western Australia 2006. Perth.

[B26] Bowden FJ, Fethers K (2008). "Let's not talk about sex": reconsidering the public health approach to sexually transmissible infections in remote Indigenous populations in Australia. Med J Aust.

[B27] Plugge E, Yudkin P, Douglas N (2009). Changes in women's use of illicit drugs following imprisonment. Addiction.

[B28] Miller ER, Bi P, Ryan P (2006). The prevalence of HCV antibody in South Australian prisoners. J Infect.

[B29] Tan JA, Joseph TA, Saab S (2008). Treating hepatitis C in the prison population is cost-saving. Hepatology.

[B30] McDonald AM, Ryan JW, Brown PR, Manners CJ, Falconer AD, Kinnear RC, Harvey WJ, Hearne PR, Banaszczyk M, Kaldor JM (1999). HIV prevalence at reception into Australian prisons, 1991-1997. Med J Aust.

[B31] Platt R (2009). Opportunity knocks: the electronic (public health) medical record. Epidemiology.

